# Longitudinal Patterns of Social Problem-Solving Skills in an Ethnically Diverse Sample of Pediatric Patients with Cancer and their Caregivers

**DOI:** 10.3390/ijerph17051581

**Published:** 2020-02-29

**Authors:** Evrosina I. Isaac, Urmila Sivagnanalingam, Andrea R. Meisman, Crista Wetherington Donewar, Linda J. Ewing, Ernest R. Katz, Anna C. Muriel, Jennifer M. Rohan

**Affiliations:** 1 Department of Pediatrics, Division of Pediatric Hematology-Oncology and Stem Cell Transplantation, Children’s Hospital of Richmond at Virginia Commonwealth University, Richmond, CA 23219, USA; sivagnanalinu@mymail.vcu.edu (U.S.);; 2 School of Medicine, Virginia Commonwealth University, Richmond, CA 23298, USA; 3 Division of Adolescent and Transition Medicine, Cincinnati Children’s Hospital Medical Center, Cincinnati, OH 45229, USA; 4 Center for Pediatric Psychiatry, Children’s Medical Center Dallas, Dallas, TX 75235, USA; 5 Department of Psychiatry, UT Southwestern Medical Center, Dallas, TX 75390, USA; 6 Department of Psychiatry, Western Psychiatric Institute and Clinic, Pittsburgh, PA 15213, USA; 7 Division of Hematology-Oncology and Blood & Marrow Transplant, Children’s Hospital Los Angeles, Los Angeles, CA 90027, USA; 8 Division of Psychosocial Oncology, Dana-Farber Cancer Institute and Boston Children’s Hospital, Boston, MA 02215, USA

**Keywords:** social problem-solving, racial differences, cancer, adherence

## Abstract

Pediatric patients with acute lymphoblastic leukemia and lymphoblastic lymphoma are prescribed a daily oral chemotherapy medication named 6-mercaptopurine. Adherence to this medication is vital for survival and decreased risk for disease relapse. Adaptive problem-solving strategies are important for adhering to this complex regimen. This manuscript examined ethnic and racial differences in social problem-solving domains (Social Problem-Solving Inventory) among patients aged 7–19 years old who were diagnosed with cancer; and, their caregivers (*N* = 139). This was a 15-month longitudinal study. We also examined differences in medication adherence based on behavioral adherence measures. Our study found significant differences between minority and non-minority reporters across multiple social problem-solving domains (*p* < 0.05). However, there were no significant differences observed for medication adherence. Our findings underscore the importance of implementing culturally sensitive interventions in clinical care that could ultimately positively impact health behaviors, interactions with healthcare providers, and long-term health outcomes.

## 1. Introduction

Acute lymphoblastic leukemia (ALL) is the most common childhood malignancy with an estimated 5900 cases diagnosed each year [1]. Hodgkin and Non-Hodgkin Lymphomas are the third most common childhood malignancy. Lymphoblastic lymphoma (LBL) affects 30% of pediatric patients diagnosed with non-Hodgkin’s lymphoma [2]. Similarities in morphology, genetics, and immunophenotypes between LBL and ALL indicated that ALL and LBL should be considered as part of a spectrum of malignant lymphoproliferative disorders [3,4,5]. Furthermore, ALL and LBL require similar cancer treatment protocols [4,5,6]. Although most children with ALL and LBL will enter remission after the induction phase, 20% will relapse within 5 years [3,4,5]. Durable remissions require a 2- to 3-year maintenance phase that includes taking daily oral 6-mercaptopurine (6-MP) to maintain disease remission [3,4,5]. Poor adherence to medication is a recognized problem and could result in negative implications, including repeated clinic visits, extended course of illness, poorly controlled symptoms, and frequent hospital admissions [4,5,7,8]. Prior studies have documented lower adherence levels among pediatric African American patients with ALL when compared to their White counterparts [9,10]; and, also lower adherence rates among Hispanic patients when compared with non-Hispanic White patients [7,11,12]. Racial and ethnic differences have also been observed in survival variability in children with ALL [11]. According to Kadan-Lottick et al. [10], Black, Hispanic, and American Indian/Alaskan Native children and adolescents with ALL have worse survival rates than White and Asian/Pacific Islander children/adolescents. Race-specific determinants to adherence and ultimately survival is poorly understood, and further investigation is needed regarding the specific factors contributing to disparities in outcomes to minimize the risk of relapse and other poor outcomes in children/adolescents with cancer. 

In order to improve medication adherence and overall health management in managing pediatric cancer, a social problem-solving framework can be utilized. The social problem-solving model describes constructive and dysfunctional styles of solving problems that may impact health and adjustment [13,14,15]. It was first described and implemented by D’Zurilla and his colleagues [14,15] to understand the relations among problem-solving ability, social competence and adaptation, stress, and various forms of maladjustment and psychopathology [16]. The social problem-solving inventory helps determine an individual’s problem-solving strengths and weaknesses so that deficits can be addressed, and treatment progress can be tracked over time. Social problem-solving abilities are correlated in predicted directions with perceptions of health and physical symptoms, secondary health complications, health-compromising behaviors, and adherence to medical regimens [16]. In addition, social problem-solving abilities have been found to influence the adjustment of family members who assume caregiving responsibilities of persons with chronic disabilities [13]. Moreover, problem-solving therapy, which is a tenet of cognitive behavioral interventions, has been shown to be effective in treating negative affectivity (e.g., depression, anxiety), enhancing parent-child relationships, and improving adherence [17,18]. 

Although prior studies have evaluated the social problem-solving model with adherence to HIV medications, depression, and spinal cord injury [13,16]; no prior research has examined social problem-solving abilities in an ethnically diverse pediatric cancer patient population with a focus on evaluating whether there are differences in problem-solving domains between minority and non-minority patients or caregivers. Determining if there are racial and ethnic differences in social problem-solving skills can lead to a better understanding of the disparities seen in health outcomes, which could lead to development of culturally sensitive interventions. A primary aim of the current study is to investigate social problem-solving domains in pediatric patients with cancer and their caregivers; and, to examine whether there are differences observed in social problem-solving abilities between minority and non-minority patients diagnosed with cancer or caregivers. Based on prior research, it is hypothesized that minority children and their caregivers would experience significantly lower social problem-solving scores as compared to non-minority peers. 

## 2. Methods 

### 2.1. Study Design

This study was a longitudinal multisite randomized controlled trial investigating a family-centered problem-solving intervention in pediatric cancer with a focus on promoting medication adherence during the maintenance phase of treatment [4,5]. Medication adherence and social problem-solving data were collected over a period of 15 months. Previous studies published from the same dataset focused on objective measures of medication adherence, including behavioral and pharmacological measures of medication adherence, and relationship to health outcomes [4,5]. The current study is focused on examining ethnic differences in parent and child social problem-solving abilities over the course of 15 months. We also examined whether there were differences in medication adherence between minority and non-minority patients.

After completing the baseline visit, participants were equally randomized into one of two groups [5]: Family Problem Solving Training Intervention (FPST, *n =* 69) or Current Psychosocial Care (CPC, *n* = 70). There were not any significant differences observed in medication adherence or social problem-solving between the control groups and intervention groups across the course of the intervention (*p* > 0.05), thus intervention group status was not included in the present analysis. Findings reported here are based on the entire sample. The specific content of the intervention is described in a previous manuscript [5]. 

### 2.2. Eligibility Criteria and Recruitment Procedures

In order to participate in the study, participants had to be prescribed a daily medication (6-mercaptopurine: 6MP), be diagnosed with ALL or LBL in remission, and have finished at least one cycle of maintenance therapy [4,5]. Patients were excluded if there was not a primary caregiver able to participate in the study, patients or caregivers were diagnosed with a chronic comorbid medical or psychiatric condition preventing them from completing study questionnaires or had known plans on moving before completing the study. In the current study, seven patients were excluded [4,5]. 

Families were first contacted by medical providers to assess interest in participation. If families agreed to be contacted by study staff, they were approached by study coordinators at each site to obtain parental permission, informed consent, and written assent (11 years and older). Verbal assent was obtained for patients younger than 10 years. Of the 171 families approached to participate [4,5], 18.7% (*n* = 32) refused participation owing to being too busy (*n* = 12), not interested in research (*n* = 19), or not having transportation (*n* = 1). There were no significant differences between those who participated and those who opted to not participate across patient age and gender (*p* > 0.05). However, it is notable that non-Hispanic Caucasian families (9.4%) chose to opt out of participation more often than Hispanic (3.5%) and non-Hispanic minority (5.8%) families (*p* = 0.01) [4,5].

### 2.3. Participants

Participants were 139 children and adolescents ages 7–19 years at baseline (*M*age = 12.3 years) who were diagnosed with either acute lymphoblastic leukemia (ALL; 96% of patients) or lymphoblastic lymphoma (LBL) (4%). Each patient had to have at least one primary caregiver participate in the study. Data were collected at six different pediatric medical centers in the United States. Institutional Review Boards were approved at each participating site. Both patients and caregivers received compensation at baseline, 6 months, and 15 months. Baseline demographic and medical characteristics are shown in Table 1.

### 2.4. Attrition Rates

Twelve patients (8.6%) dropped out of the study due to disease relapse (*n* = 9, 75%), maintenance therapy finishing before study completion (*n* = 1, 8.3%), and transfer of care to another hospital (*n* = 2, 16.7%). Of the families who did not complete the study, 8.3% (*n* = 1) completed baseline, 33.3% (*n* = 4) completed 3 months, 25% (*n* = 3) completed 6 months, 25% (*n* = 3) completed 9 months, and 8.3% (*n* = 1) completed 12 months [4,5]. 

### 2.5. Measures

*Social Problem-Solving Inventory (SPSI).* The SPSI is a self-report instrument that can be administered to children, adolescents, and adults. There are different versions of the measure based on respondent age. It is notable that the self-report adult version of the Social Problem-Solving Inventory that caregivers completed had different scales than the adolescent inventory that patients completed. All children and adolescents in the present study completed the Social Problem-Solving Inventory for Adolescents (SPSI-A; 64 items), which has three primary subscales or domains: Automatic Process, Problem Orientation, and Problem-Solving Skills. The latter two subscales also have three and four subscales respectively. The Problem Orientation Scale is made up of the Cognition, Emotion, and Behavior subscales. The Problem-Solving Skills Scale is made up of the Problem Identification, Alternative Generation, Consequence Prediction, and Implementation/ Evaluation/Reorganization subscales. Based on recommendations for capturing problem-solving subgroups [14,15], problem-solving subgroups were created based on data from normative samples and the current sample. Scores within the 25th percentile were considered “ineffective problem-solvers,” scores within the >25th to 75th percentiles were considered “average problem-solvers,” and scores > 75th percentile were considered “effective problem-solvers.” See Supplemental Table S1 for an overview of each domain. 

Caregivers completed the Social Problem-Solving Inventory–Revised: Long questionnaire (SPSI-R:L; 52 items), which is a self-reported measure of their own problem-solving abilities. The SPSI-R:L has eight subscales: Positive Problem Orientation, Negative Problem Orientation, Impulsivity/Carelessness Style, Avoidance Style, Problem Definition and Formulation, Generation of Alternative Solutions, Decision Making, and Solution Implementation and Verification. The last four subscales of this measure yield the *Rational Problem-Solving* score. This revised survey was developed to look at the constructive dimensions of problem-solving, as well as, the dysfunctional dimensions of problem-solving; and, how problem orientation and problem-solving can have both functional as well as dysfunctional elements. All items are scored on a 5-point scale ranging from *“Not at All True of Me”* to *“Extremely True of Me.”* Based on recommendations for scoring and determining problem-solving subgroups [14,15], problem-solving subgroups were created using the following dimensions: standard scores ≥ 145 were considered “extremely above the norm group average,” scores of 130–144 were considered “very much above the norm group average,” scores of 115–129 were considered “above the norm of the group average,” scores of 86–114 were considered “the norm group average,” scores of 71–85 were considered “below the norm of the group average,” scores of 56–70 were considered “very much below the norm of the group average,” and scores < 56 were considered “extremely below the norm of the group average.” See Supplemental Table S2 for an overview of each problem-solving domain in the SPSI-R:L.

### 2.6. Medication Adherence

The Medication Event Monitoring System (MEMS Cap) [19] is a device that looks similar to a prescription bottle that you would get at the pharmacy. The bottle cap has a computer chip that reads the time and date that the bottle was opened. The computer software comes with a reader which downloads information from the cap. On the software, events can be edited to count for medication breaks, multiple doses a day, or doses on only certain days a week. This makes the software and device adaptable to different medication schedules. The MEMS Cap has been validated to be an accurate representation of medication adherence and has been validated with pharmacological measures of medication adherence in pediatric cancer [5,7]. In this study, there was no significant difference between adherence reported by the MEMS Cap and adherence as determined by metabolite 6MP levels [5]. Adherence was downloaded at quarterly intervals across the 15 months of the study.

## 3. Results

### 3.1. Social Problem-Solving Inventory

A chi-squared analysis was done to analyze ethnic and racial differences across social-problem solving groups for the child and caregiver reporters. Additionally, mean group comparisons were conducted to determine whether there were differences in social problem-solving abilities between minority versus non-minority groups for both patient and caregiver reporters. Prior to conducting group-based analyses, baseline SPSI scores were correlated with SPSI scores obtained at 6- and 15-months using bivariate Pearson correlations. On all subscales, baseline, 6, and 15-month scores were significantly correlated with moderate to strong correlations observed (r=0.30 to 0.80). Results of patient versus caregiver problem-solving skills are reported below. 

### 3.2. Social Problem-Solving Inventory: Patient Reporters

A chi-squared analysis was done to analyze ethnic and racial differences in social problem-solving groups (ineffective, average, effective problem-solving abilities) between minority versus non-minority patients with cancer. There was a statistically significant association between non-minority and minority patients in the subscales of Cognition (χ^2^ = 28.640; *p* = 0.026) at six months, Alternative Generation (χ^2^ = 8.782; *p* = 0.012) at baseline, Implementation (χ^2^ = 7.886; *p* = 0.019) at 15 months, and Problem Orientation (χ^2^ = 7.120; *p* = 0.028) at six months. As shown in Supplemental Table S3, at baseline, the highest percentage of patients (42–53%) fell into the average problem-solvers group in each of the subscales with the exception of the Cognition scale, which had an equal distribution between ineffective and average problem-solvers (35.8%). At 6 months, a similar pattern was seen across all subscales (42–53%) with the exception of Cognition where the highest percentage of patients, 37.7%, were in the effective problem-solvers category. At 15 months, the highest percentage of patients were in the average problem-solvers category across all domains (38–53%) except for Evaluation where the highest percentage, 36.1%, of patients fell into the effective problem-solvers category.

To further analyze the mean differences between non-minority and minority patients, an independent samples t-test was performed for each domain. Descriptive statistics are shown in Table 2. At baseline, minority patients had significantly lower scores (1.90 ± 1.11) than their non-minority counterparts (2.32 ± 0.97) on the Alternative Generation subscale, *t*(132) = 2.32, *p* = 0.02. At six months, minority patients had significantly lower scores on all three subscales of the Problem Orientation scale (Cognition: *t*(128) = 2.19, *p* = 0.03; Emotion: *t*(128) = 2.31, *p* = 0.02; Behavior: *t*(128) = 2.13, *p* = 0.04), the total Problem Orientation scale (*t*(128) = 2.95, *p* = 0.004), two of the Problem-Solving Skills subscales (Alternative Generation: *t*(128) = 2.26, *p* = 0.03; Evaluation: *t*(128) = 2.48, *p* = 0.01), and the total problem-solving score (*t*(128) = 2.04, *p* = 0.04). In contrast, at the 15 month time point, minority patients had significantly higher scores (2.30 ± 1.12) on the Implementation subscale than non-minority patients (1.85 ± 1.09), *t*(117) = −2.29, *p* = 0.04.

### 3.3. Social Problem-Solving Inventory: Caregiver Reporters 

For parent-reported social problem-solving, a chi-squared analysis was completed to examine the relationship between ethnicity/race group status and social problem-solving abilities. For adult reporters, there are seven problem-solving groups ranging from *“extremely above norm group average”* to *“extremely below norm group average*.” In contrast to child/adolescent reporters, there were significant differences in parent-reported problem-solving abilities at all three visits for the Problem Definition and Formulation subscale, which had significant differences observed between non-minority and minority reporters at: baseline: χ^2^ = 12.46; *p* = 0.03, 6 months: χ^2^ = 13.82; *p* = 0.017, and 15 months: χ^2^ = 14.23; *p* = 0.014. At baseline, there also was a significant difference in Generation of Alternative Solutions between non-minorities and minorities (χ^2^ = 11.44; *p* = 0.02). At 6 months, there were significant differences in the Decision Making subscale (χ^2^ = 17.98; *p* = 0.001) and the Rational Problem Solving scale (χ^2^ = 14.72; *p* = 0.012) between minorities and non-minorities. For Solution Implementation and Verification, there were significant differences between minorities and non-minorities at baseline (χ^2^ = 12.23; *p* = 0.032) and 6 months (χ^2^ = 13.14; *p* = 0.011), but not at 15 months (*p* > 0.05). Similarly, there were significant differences observed in Negative Problem Orientation at 6 months (χ^2^ = 10.48; *p* = 0.033) and at 15 months: χ^2^ = 12.46; *p* = 0.006; and, in Avoidance Style at 6 months (χ^2^ = 12.81; *p* = 0.012) and 15 months (χ^2^ = 9.70; *p* = 0.021). Supplemental Table S4 provides the percentages of reporters within each category. Similar to patient-reporters, the highest percentage of caregivers fell into the norm group average for all subscales at baseline (50–66%) and 15 months (47–74%). At 6 months, a similar pattern was seen (50–69%), however for Impulsivity/Carelessness Style, the highest percentage of caregivers, 48.1%, was seen in the below norm group average.

We further analyzed the mean differences in problem-solving scores between minority and non-minority patients using an independent samples t-test. Descriptive statistics are shown in Table 3. At 6 months, non-minority caregivers had significantly higher scores in two of the Rational Problem Solving subscales (Problem Definition and Formulation: *t*(131) = 2.59, *p* = 0.01); Decision Making: *t*(130) = 2.24, *p* = 0.03) as well as in Positive Problem solving (*t*(131) = 2.010, *p* = 0.038). At 15 months, non-minority caregivers had significantly higher scores in Negative Problem Orientation (*t*(119) = 3.53, *p* = 0.001), Avoidance Style (*t*(119) = 3.06, *p* = 0.003), as well as three of the Rational Problem Solving subscales (Problem Definition and Formulation: *t*(118) = 2.04, *p* = 0.044, Generation of Alternative Solutions: *t*(117) = 2.11, *p* = 0.037; and Decision Making: *t*(119) = 2.08, *p* = 0.04).

### 3.4. Medication Adherence

There were no significant differences observed in medication adherence across the 15-month monitoring period (*p* > 0.05). Supplemental Figure S1 shows the adherence rates for minority versus non-minority patients. 

## 4. Discussion

Contrary to Bhatia et al. [7], we found no significant difference in longitudinal adherence rates between minority and non-minority patient populations (Supplemental Figure S1). In fact, in the present study, there were several points in time where minority patients had higher adherence rates than non-minority patients. The contradicting findings in our study relative to previous studies could potentially be explained by the provider-patient relationship. Hispanic patients often report that they prefer health care providers who are warm, personal, and treat them with dignity [11,20,21,22,23]. In addition, prior research has suggested that providers do not always use interpreters during medical visits and that some providers may use the patient or the parent/caregiver as the translator, which puts the parents and/or child in a reversed power and authority position, which is contradictory to cultural norms [11,20,21,22,23,24]. It also is important to include the entire family in making health care decisions as familialism is a universal value in the Hispanic community [25,26]. Furthermore, therapeutic rapport is a strong predictor of treatment adherence and ultimately health outcomes [11,20,21,22,23,24]. If rapport is strong, there is an increased probability that adherence will be high. If rapport is weak, there is an increased probability that adherence will be low. As a result, it is possible that observed differences in treatment adherence may partially be related to the interactions between providers and their patients/families. Thus, future research should examine parent-child-provider relationships and its effects on health behaviors and treatment outcomes. 

To our knowledge, no prior research has examined ethnic/racial differences in social problem-solving abilities in the pediatric oncology population and their caregivers, specifically using the Social Problem-Solving Inventory as the unit of measure. Our study found significant differences in child/adolescent-reported and caregiver-reported social problem-solving scores when looking at ethnicity/race categorized as a binary variable (i.e., non-minority versus minority). In the present study, minority patients had significantly higher scores than non-minority patients on the Implementation scale at 15 months, which suggests that minority patients are more likely to be able to implement a selected solution by creating a strategic plan of action. Progress is evaluated by successful goal attainment. If the problem is not solved, the individual will re-organize their process and re-evaluate another solution until the problem is successfully solved [27].

On the other hand, non-minority patients had higher scores than minority patients across all other problem-solving domains. At baseline, non-minority patients had significantly higher scores in Alternative Generation suggesting that non-minority patients were able to more readily devise different solutions to identified problems. Similarly, at 6 months, non-minority patients had significantly higher scores than minority patients in Cognition, Emotion, Behavior, Problem Orientation, Alternative Generation, Evaluation, and overall problem-solving. These findings suggest that non-minority patients have confidence in their intellectual capacities to engage in the problem-solving process, have heightened emotions when faced with problems that need to be solved, and an increased willingness to approach the problem in question. Non-minority patients also had higher scores on the Problem Orientation Scale, i.e., the motivational component of social problem-solving, suggesting that non-minority patients have increased motivation when addressing real-life problems and will often utilize individual beliefs, attitudes, and values to solve problems. 

When looking at caregiver reports at 6 months, non-minority caregivers had higher scores than minority caregivers in Problem Definition/Formulation, Decision Making, and Positive Problem Solving. This finding suggests that these caregivers are not only trying to understand the problem by looking for facts and setting realistic goals to solve problems, but also anticipate different consequences of different solutions; and, thus will come to an effective decision on how to solve the problem. In addition, non-minority caregivers will often perceive their problems as solvable challenges. At 15 months, non-minority caregiver respondents had higher scores than minority caregivers in Negative Problem Orientation, Avoidance Style, Problem Definition/Formulation, Generation of Alternative Solutions, and Decision Making. These results suggest that non-minority caregivers may have a more dysfunctional cognitive emotional set where they view problems as being a threat to them or may become more easily frustrated when problems are faced. Similarly, they may procrastinate or be completely passive when faced with problems. On the other hand, these caregivers may use more adaptive problem-solving skills like trying to clarify and understand the problem by gathering facts, focusing on specific and concrete information, identifying obstacles, and setting a specific goal for problem-solving while also generating as many alternative solutions as possible. Finally, these individuals will likely try to predict the positive and negative consequences of each possible solution, considering both the immediate and long-term consequences as well as the personal and social consequences. Our findings are remarkably different than what Kasckow et al. [28] found when examining social problem-solving abilities between Caucasian and African American patients. These authors found no significant differences among these subgroups. 

Although the present study did not find any significant relationships between medication adherence and social problem-solving, it is notable that problem-solving abilities could have an impact on other parts of the patient’s self-management behaviors, other health behaviors, and ultimately health outcomes. For example, problem-solving may impact clinic attendance (e.g., do patients come to clinic on time or are they always late for their medical appointments?). Furthermore, dysfunctional problem-solving within the family system could cause problems between the child-caregiver dyad, which could increase family conflict and lead to worse health behaviors and, ultimately, worse health outcomes over time [4,5,7,10,11,12,22,23,24].

There are limitations of the present study that should be considered in future research. As with any prescription bottle, just because a prescription bottle is opened or a pill is removed from the device, this does not necessarily correlate with the patient ingesting the medication. That said, in a previous study investigating the relationship between metabolites of 6MP and behavioral adherence (MEMs Caps) that was conducted in the same patient cohort as the present study [5], there was no significant difference observed between adherence reported by the MEMS caps and 6MP metabolite levels. This finding suggests that patients were truly taking the medicine after opening the bottle. However, there was variability in a smaller cohort of patients who had incongruent adherence results between these two adherence measures. While this previous finding strengthens the feasibility for using electronic monitoring devices in clinical settings, a larger study should be completed to further validate the MEMS caps as an accurate measure for tracking medication adherence. Furthermore, in the present study, information on parent-report of their child’s problem-solving skills was not obtained. It is notable that the adult report of the Social Problem-Solving Inventory, which caregivers completed, has different subscales than the adolescent inventory, which ultimately prevents us from making a direct comparison of child/adolescent and parent problem-solving abilities. Future research should examine not only parent assessment of their own problem-solving, but also parent-report of adolescent problem-solving. 

Our findings underscore the importance of implementing culturally relevant interventions in routine clinical care. While there were no significant differences observed in medication adherence in the current sample, our findings demonstrated differences in problem-solving skills between minority and non-minority subgroups, which could ultimately impact health behaviors, interactions with healthcare providers, and ultimately long-term health outcomes. Future research investigating the specific relationship between problem-solving abilities, health behaviors like medication adherence, and health outcomes while taking into consideration cultural differences is imperative for developing clinically effective interventions.

## 5. Conclusions

Our findings underscore the influence of race and ethnicity factors on psychological and health outcomes in pediatric cancer patients. Our findings highlight how a social problem-solving framework can be utilized to improve medication adherence and overall health management in managing pediatric cancer. Utilizing social problem-solving inventories in routine medical care will help determine problem-solving strengths and weaknesses so that deficits can be addressed. Determining if there are racial and ethnic differences in social problem-solving skills can lead to a better understanding of the disparities seen in health outcomes. Our findings highlight the importance of developing culturally sensitive interventions that utilize a combination of problem-solving therapy techniques and cognitive behavioral interventions to treat psychological distress, enhance parent-child relationships, and improve adherence and other health behaviors.

## Figures and Tables

**Table 1:** Demographic and Medical Characteristics of Baseline Sample (*N* = 139).

Baseline Demographic and Medical Variables	M ± SD or *n* (%)
Patient age at baseline (years), M ± SD	12.29 years ± 3.44
Type of Cancer Diagnosis, n (%)	
ALL	133 (95.7)
LBL	6 (4.3)
Duration of Cancer Diagnosis at Baseline (Years), M ± SD	1.29 years ± 0.35
Child’s Gender, n (%)	
Male	94 (67.6)
Female	45 (32.4)
Child’s Ethnicity/Race, n (%)	
Non-Hispanic, Caucasian	75 (54.0)
Non-Hispanic, Minority	16 (11.5)
Hispanic	48 (34.5)
Primary Caregiver’s Marital Status, n (%)	
Married	96 (69.1)
Not Married	43 (30.9)
Household Composition, n (%)	
One caregiver household	45 (32.4)
Two caregiver household	94 (67.6)

**Table 2:** Mean Scores for Social Problem-Solving Skills Inventory (SPSI-A): Overall Sample vs. Minority vs. non-Minority Patient Reporters.

Subscale	Overall	Minority	Non-Minority		Overall	Minority	Non-Minority	Overall	Minority	Non-Minority
	Baseline (M ± SD)			6 months (M ± SD)				15 months (M ± SD)		
APS	2.45 ± 0.88	2.37 ± 0.93	2.53 ± 0.84		2.45 ± 0.88	2.37 ± 0.90	2.52 ± 0.85	2.60 ± 0.81	2.53 ± 0.73	2.65 ± 0.86
COG	1.67 ± 0.45	1.61 ± 0.47	1.72 ± 0.43		1.68 ± 0.44	1.58 ± 0.47	1.75 ± 0.40 *	1.70 ± 0.43	1.69 ± 0.42	1.72 ± 0.44
EMO	0.77 ± 0.51	0.78 ± 0.48	0.76 ± 0.54		0.80 ± 0.51	0.69 ± 0.45	0.89 ± 0.53 *	0.74 ± 0.52	0.73 ± 0.50	0.75 ± 0.54
BEH	0.72 ± 0.51	0.76 ± 0.53	0.68 ± 0.50		0.77 ± 0.54	0.66 ± 0.49	0.86 ± 0.56 *	0.82 ± 0.52	0.80 ± 0.51	0.83 ± 0.53
POS	1.05 ± 0.33	1.05 ± 0.32	1.05 ± 0.35		1.08 ± 0.38	0.98 ± 0.35	1.17 ± 0.38 **	1.08 ± 0.34	1.07 ± 0.32	1.10 ± 0.35
PID	1.91 ± 0.99	1.87 ± 1.02	1.94 ± 0.96		1.81 ± 0.92	1.65 ± 0.89	1.95 ± 0.92	1.91 ± 0.99	2.00 ± 0.93	1.84 ± 0.91
ALT	2.12 ± 1.06	1.90 ± 1.11	2.32 ± 0.98 *		2.07 ± 1.02	1.85 ± 1.00	2.25 ± 1.00 *	2.12 ± 0.97	2.11 ± 0.95	2.12 ± 0.99
CON	1.98 ± 1.05	1.97 ± 1.13	2.00 ± 0.98		1.99 ± 1.00	1.82 ± 0.95	2.12 ± 1.02	2.02 ± 1.03	2.07 ± 1.06	1.98 ± 1.02
IMP	1.98 ± 1.17	2.02 ± 1.25	1.95 ± 1.01		2.10 ± 1.14	2.14 ± 1.16	2.07 ± 1.13	2.05 ± 1.09	2.30 ± 1.12	1.85 ± 1.04 *
EVL	2.41 ± 0.96	2.34 ± 1.01	2.47 ± 0.91		2.30 ± 0.96	2.07 ± 1.02	2.49 ± 0.87 *	2.41 ± 0.96	2.51 ± 1.02	2.33 ± 0.91
REO	2.26 ± 1.08	2.17 ± 1.13	2.33 ± 1.02		2.24 ± 0.95	2.08 ± 0.95	2.36 ± 0.94	2.31 ± 1.02	2.34 ± 1.06	2.29 ± 0.99
PSSS	2.11 ± 0.90	2.04 ± 0.97	2.17 ± 0.83		2.08 ± 0.87	1.94 ± 0.85	2.21 ± 0.88	2.14 ± 0.88	2.22 ± 0.90	2.07 ± 0.86
TOT	1.87 ± 0.56	1.82 ± 0.60	1.92 ± 0.51		1.87 ± 0.57	1.76 ± 0.59	1.96 ± 0.54 *	1.94 ± 0.53	1.94 ± 0.53	1.94 ± 0.54

Note: The abbreviated subscales in Table 2 and Supplemental Table S3 represent the following subscales in the SPSI-A: Automatic Process Scale (APS); Problem Orientation Scale (POS), which is the Cognition (COG), Emotion (EMO), and Behavior (BEH) subscales. The Problem-Solving Skills Scale (PSSS), which is the Problem Identification (PID), Alternative Generation (ALT), Consequence Prediction (CON), and Implementation/Evaluation/Reorganization (IMP/EVL/REO). TOT = Total Problem-Solving Score. Significant differences between minority versus non-minority groups are noted by * *p* < 0.05 or ** *p* < 0.01.

**Table 3:** Mean Scores for Social Problem-Solving Skills Inventory: Overall Sample vs. Minority vs. non-Minority Caregiver Reporters.

Subscale	Overall	Minority	Non-Minority	Overall	Minority	Non-Minority	Overall	Minority	Non-Minority
	Baseline			6 months			15 months		
PPO	91.71 ± 17.44	90.91 ± 19.37	92.40 ± 15.71	92.00 ± 17.24	88.58 ± 17.69	94.81 ± 16.46*	94.10 ± 17.29	91.74 ± 17.77	95.94 ± 16.82
NPO	94.37 ± 15.47	93.19 ± 17.83	95.39 ± 13.17	92.13 ± 15.50	89.32 ± 16.79	94.44 ± 14.06	90.05 ± 13.01	85.53 ± 13.32	93.57 ± 11.70**
RPS	93.31 ± 19.15	91.95 ± 22.18	94.47 ± 16.19	92.89 ± 17.94	89.65 ±20.65	95.60 ± 14.95	93.50 ± 19.02	89.74 ± 21.21	96.43 ± 16.69
ICS	90.96 ± 15.06	91.31 ± 17.63	90.65 ± 12.58	88.36 ± 12.78	86.12 ± 13.46	90.21 ± 11.96	88.14 ± 11.32	87.85 ± 13.72	88.37 ± 9.12
AS	94.58 ± 13.93	92.97 ± 15.36	95.96 ± 12.52	93.60 ± 12.34	91.93 ± 14.12	94.97 ± 10.56	93.43 ± 10.65	90.19 ± 10.45	95.96 ± 10.18
PDF	93.16 ± 18.70	91.20 ± 21.33	94.83 ± 16.08	93.06 ± 18.30	88.62 ± 20.13	96.71 ± 15.88*	93.96 ± 18.88	90.06 ± 21.65	97.04 ± 15.86**
GAS	94.13 ± 17.04	92.42 ± 19.21	95.49 ± 15.08	94.68 ± 15.89	92.05 ± 16.98	96.81 ± 14.73	94.82 ± 17.40	90.98 ± 18.90	97.69 ± 15.72*
DM	93.62 ± 17.88	91.11 ± 20.09	95.76 ± 15.56	93.20 ± 18.49	89.32 ± 21.22	96.43 ± 15.26*	93.75 ± 19.03	89.74 ± 20.84	96.88 ± 17.00*
SIV	94.93 ± 18.38	95.16 ± 20.98	94.75 ± 16.02	94.99 ± 17.38	93.92 ± 20.64	95.89 ± 14.21	94.86 ± 17.43	93.13 ± 18.81	96.21 ± 16.29*
SPSI	101.07 ± 13.94	101.31 ± 14.76	100.87 ± 12.29	102.42 ± 13.37	102.67 ±14.21	102.22 ± 12.73	103.77 ± 12.68	104.81 ± 13.59	102.96 ± 11.96

Note: The abbreviated subscales in Table 3 and Supplemental Table S4 represent the following subscales in the SPSI-R:L: Positive Problem Orientation (PPO); Negative Problem Orientation (NPO), Relational Problem-Solving Scale (RPS), Impulsivity-Carelessness Scale (ICS), Avoidance Style Scale (AS), Problem Definition/Formulation (PDF), Generation of Alternative Solutions (GAS), Decision Making (DM), Solution Implementation/Verification (SIV), Total Problem-Solving Skills Score (SPSI). Significant differences between minority versus non-minority groups are noted by * *p* < 0.05 or ** *p* < 0.01.

## Supplementary Materials

The following are available online at https://www.mdpi.com/1660-4601/17/5/1581/s1, Figure S1: Medication Adherence Rates between Minority versus Non-Minority Patients, Table S1: Overview of the SPSI-A Subscales, Table S2: Overview of the SPSI-R:L Subscales, Table S3: Percentage of Patients in each Problem-Solving Category for each Subscale (SPSI-A), Table S4: Percentage of Caregivers in each Problem-Solving Category for each Subscale (SPSI-R:L).
