# Supplementary material for: Longitudinal Patterns of Social Problem-Solving Skills in an Ethnically Diverse Sample of Pediatric Patients with Cancer and their Caregivers
Source: Int J Environ Res Public Health. 2020 Feb 29;17(5):1581. doi: 10.3390/ijerph17051581 (PMC7084745; doi:10.3390/ijerph17051581)
Supplement: Supplementary file 1 [file ijerph-17-01581-s001.pdf]

Supplementary Tables and Figures

Table S1. Overview of the SPSI-A Subscales.

Domain	Scale Abbreviation	Definition of Social Problem-Solving Domain
Automatic Process	APS	Individual will automatically or over time apply strategies that were previously successful in solving similar conflicts.
Evaluation Process	EVL	This process is used when an individual cannot solve the problem by applying automatic or habitual strategies that were successful in solving other problems. This process utilizes two separate strategies: problem-orientation and problem-solving skills.
Problem Orientation	POS	This is the motivational component of social problem-solving and addresses individual beliefs, attitudes, and values regarding the social problem-solving process for solving real-life problems.
Cognition	COG	Person has confidence in his/her intellectual capacities to engage in the social problem-solving process
Emotion	EMO	Feelings that a person experiences when the person is faced with the need to solve a problem
Behavior	BEH	Willingness of a person to approach the problem rather than avoiding the process
Problem-Solving Skills	PSSS	The combination of all of these skills together are critical for the success of the social problem-solving model.
Problem Identification	PID	Identify that a problem exists through the use of cues, attitudes, emotions, behaviors, and physiologic responses.
Alternative Generation	ALT	This is the cognitive skill of brain storming or free-associating by generating a list of alternative options or solutions for the identified problem.
Consequence Prediction	CON	Individuals attempt to predict consequences of each solution, including weighing the advantages and disadvantages of each solution.
Implementation/Evaluation/ Reorganization	IMP/EVL/ REO	Individual will implement the selected solution by creating a strategic plan of action. Progress is evaluated by successful goal attainment. If the problem is not solved, the individual will re-organize their process and re-evaluate another solution until the problem is successfully solved.

Table S2. Overview of the SPSI-R:L Subscales.

Domain	Scale Abbreviation	Definition of Social Problem-Solving Domain
Positive Problem Orientation	PPO	Individuals with higher scores are likely to appraise a problem as a challenge rather than a threat and will believe that their problem is solvable
Negative Problem Orientation	NPO	Measures a dysfunctional or inhibitive cognitive-emotional set. Higher scores are likely to indicate a problem as a significant threat to well-being, doubt personal abilities to solve problems successfully, and become frustrated or upset when confronted with problems
Relational Problem-Solving Scale	RPS	Assesses the rational, deliberate, and systematic application of effective problem-solving strategies. Higher scores indicate individuals who carefully and systematically solve problems
Problem Definition/ Formulation	PDF	Higher scores indicate individuals will try to clarify and understand the problem by gathering facts, focusing on specific and concrete information, identifying obstacles, and setting a specific goal for problem-solving
Generation of Alternative Solutions	GAS	Higher scores indicate individuals will focus on their problem-solving goals and attempt to generate as many alternative solutions as possible
Decision Making	DM	Higher scores indicate that individuals will try to predict the positive and negative consequences of each solution alternative, considering both immediate and long-term consequences as well as personal and social consequences
Solution Implementation/ Verification	SIV	Higher scores indicate individuals will practice their chosen solutions to increase their chances of success and will evaluate how much the situation and their emotions have changed for the better. If the outcome is not satisfactory, individuals will re-evaluate their solutions and will attempt problem-solving process again
Impulsivity-Carelessness Scale	ICS	Higher scores indicate individuals who consider only a few solution alternatives; impulsively going with the first idea that comes to mind
Avoidance Style Scale	AS	Higher scores indicate more likely to avoid problems rather than confront them or procrastinate with solving problems

Table S3. Percentage of Patients in each Problem-Solving Category for each Subscale (SPSI-A).

BASELINE	APS	COG	EMO	BEH	PID	ALT	CON	IMP	EVL	REO
Ineffective Problem-Solvers	26.9	35.8	18.7	32.1	25.4	29.1	30.6	27.6	27.6	22.4
Average Problem-Solvers	46.3	35.8	53.0	47.0	48.5	42.5	42.5	48.5	43.3	50.7
Effective Problem-Solvers	26.9	28.4	28.4	20.9	26.1	28.4	26.9	23.9	29.1	26.9
**6 MONTH**	**APS**	**COG**	**EMO**	**BEH**	**PID**	**ALT**	**CON**	**IMP**	**EVL**	**REO**
Ineffective Problem-Solvers	29.2	31.5	20	33.8	22.3	25.4	27.7	23.1	25.4	26.9
Average Problem-Solvers	42.3	30.8	51.5	42.3	53.8	51.5	46.9	50.8	45.4	41.5
Effective Problem-Solvers	28.5	37.7	28.5	23.8	23.8	23.1	25.4	26.2	29.2	31.5
**15 MONTH**	**APS**	**COG**	**EMO**	**BEH**	**PID**	**ALT**	**CON**	**IMP**	**EVL**	**REO**
Ineffective Problem-Solvers	26.7	29.2	19.3	30.3	27.7	25.2	32.8	20.2	28.6	27.7
Average Problem-Solvers	49.2	38.3	52.9	43.7	47.1	49.6	39.5	52.9	35.3	43.7
Effective Problem-Solvers	24.2	32.5	27.7	26.1	25.2	25.2	27.7	26.9	36.1	28.6

Table S4. Percentage of Caregivers in each Problem-Solving Category for each Subscale (SPSI-R:L).

BASELINE	PPO	NPO	RPS	ICS	AS	PDF	GAS	DM	SIV
Extreme Above Norm Group Average	0.0	0.0	0.0	0.7	0.7	0.0	0.0	0.0	0
Very Much Above Norm Group	0.7	2.2	1.4	1.4	3.6	1.4	0.0	1.4	2.9
Above Norm Group Average	10.1	10.8	11.5	6.5	5.8	11.5	10.1	10.8	11.6
Norm Group Average	49.6	54.0	52.5	51.1	66.2	51.8	59.7	54.7	52.2
Below Norm Group Average	31.7	30.9	23.0	36.7	23.0	23.7	17.3	20.9	25.4
Very Much Below Norm Group	5.8	2.2	10.1	3.6	0.7	9.4	9.4	11.5	6.5
Extreme Below Norm Group Average	2.2	0.0	1.4	0.0	0.0	2.2	3.6	0.7	1.4
**6 MONTH**	**PPO**	**NPO**	**RPS**	**ICS**	**AS**	**PDF**	**GAS**	**DM**	**SIV**
Extreme Above Norm Group Average	0.0	0.0	0.0	0.0	0.0	0.0	0.0	0.0	0
Very Much Above Norm Group	0.8	3.8	1.5	0.0	0.8	1.5	0.8	3.0	2.3
Above Norm Group Average	9.8	5.3	8.3	5.3	6.0	9.8	9.8	6.8	12.9
Norm Group Average	56.4	50.4	59.1	42.9	69.2	51.9	60.2	55.3	54.5
Below Norm Group Average	23.3	38.3	18.9	48.1	23.3	24.1	20.3	23.5	22.7
Very Much Below Norm Group	7.5	2.3	10.6	3.8	0.8	10.5	8.3	11.4	7.6
Extreme Below Norm Group Average	2.3	0.0	1.5	0.0	0.0	2.3	0.8	0.0	0
**15 MONTH**	**PPO**	**NPO**	**RPS**	**ICS**	**AS**	**PDF**	**GAS**	**DM**	**SIV**
Extreme Above Norm Group Average	0.0	0.0	0.0	0.0	0.0	0.0	0.0	0.0	0
Very Much Above Norm Group	0.0	0.0	3.3	0.0	0.8	1.7	0.8	0.8	1.7
Above Norm Group Average	11.6	3.3	9.1	3.3	5.8	12.5	13.2	13.2	11.6
Norm Group Average	57.9	51.2	53.7	51.2	73.6	49.2	57.0	47.1	55.4
Below Norm Group Average	20.7	41.3	23.1	42.1	19.8	25.8	17.4	24.8	24
Very Much Below Norm Group	9.9	4.1	8.3	3.3	0.0	8.3	9.1	12.4	6.6
Extreme Below Norm Group Average	0.0	0.0	2.5	0.0	0.0	2.5	2.5	1.7	0.8

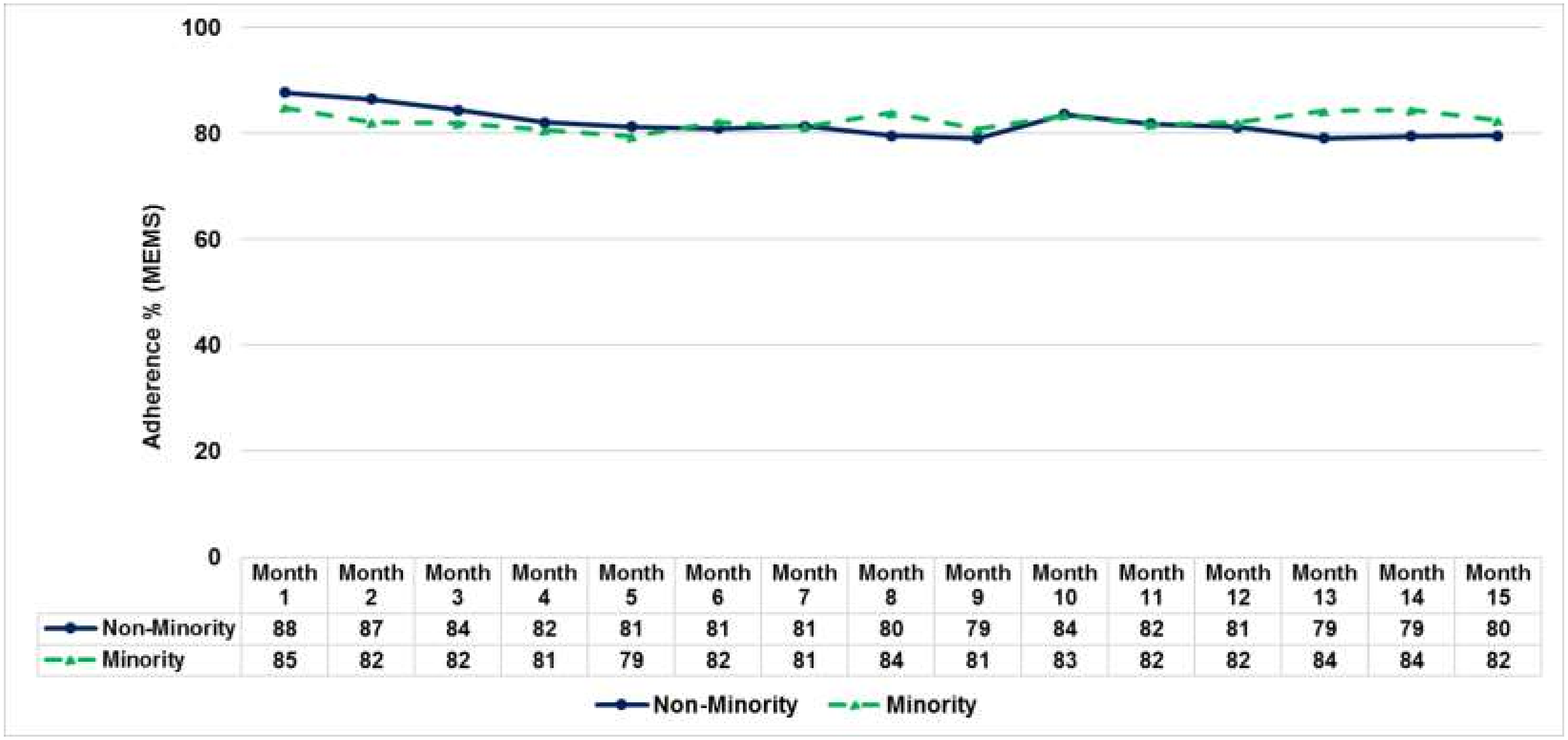

	Month 1	Month 2	Month 3	Month 4	Month 5	Month 6	Month 7	Month 8	Month 9	Month 10	Month 11	Month 12	Month 13	Month 14	Month 15
Non-Minority	88	87	84	82	81	81	81	80	79	84	82	81	79	79	80
Minority	85	82	82	81	79	82	81	84	81	83	82	82	84	84	82

Figure S1. Medication Adherence Rates between Minority versus Non-Minority Patients.
